# Identification of immunodominant T cell epitopes induced by natural Zika virus infection

**DOI:** 10.3389/fimmu.2023.1247876

**Published:** 2023-08-29

**Authors:** Christopher S. Eickhoff, Krystal A. Meza, Frances E. Terry, Chase G. Colbert, Azra Blazevic, Andres H. Gutiérrez, E. Taylor Stone, James D. Brien, Amelia K. Pinto, Hana M. El Sahly, Mark J. Mulligan, Nadine Rouphael, Maria L. Alcaide, Kay M. Tomashek, Chris Focht, William D. Martin, Leonard Moise, Anne S. De Groot, Daniel F. Hoft

**Affiliations:** ^1^ Department of Internal Medicine, Saint Louis University, Division of Infectious Diseases, Allergy, and Immunology, Saint Louis, MO, United States; ^2^ EpiVax, Inc., Providence, RI, United States; ^3^ Department of Molecular Microbiology and Immunology, Saint Louis University, Saint Louis, MO, United States; ^4^ Department of Molecular Virology and Microbiology, Baylor College of Medicine, Houston, TX, United States; ^5^ New York University Grossman School of Medicine, Division of Infectious Diseases and Immunology, New York, NY, United States; ^6^ Emory University School of Medicine, Division of Infectious Diseases, Department of Internal Medicine, Atlanta, GA, United States; ^7^ University of Miami, Division of Infectious Diseases, Miller School of Medicine, Miami, FL, United States; ^8^ Division of Microbiology, Immunology and Infectious Diseases, National Institute of Allergy and Infectious Diseases, National Institutes of Health (NIH), Bethesda, MD, United States; ^9^ The Emmes Company, LLC., Rockville, MD, United States; ^10^ University of Georgia Center for Vaccines and Immunology, Athens, GA, United States

**Keywords:** Zika virus, T cell, immunoinformatics, epitopes, immunodominance, EpiMatrix, JanusMatrix

## Abstract

Zika virus (ZIKV) is a flavivirus primarily transmitted by *Aedes* species mosquitoes, first discovered in Africa in 1947, that disseminated through Southeast Asia and the Pacific Islands in the 2000s. The first ZIKV infections in the Americas were identified in 2014, and infections exploded through populations in Brazil and other countries in 2015/16. ZIKV infection during pregnancy can cause severe brain and eye defects in offspring, and infection in adults has been associated with higher risks of Guillain-Barré syndrome. We initiated a study to describe the natural history of Zika (the disease) and the immune response to infection, for which some results have been reported. In this paper, we identify ZIKV-specific CD4+ and CD8+ T cell epitopes that induce responses during infection. Two screening approaches were utilized: an untargeted approach with overlapping peptide arrays spanning the entire viral genome, and a targeted approach utilizing peptides predicted to bind human MHC molecules. Immunoinformatic tools were used to identify conserved MHC class I supertype binders and promiscuous class II binding peptide clusters predicted to bind 9 common class II alleles. T cell responses were evaluated in overnight IFN-γ ELISPOT assays. We found that MHC supertype binding predictions outperformed the bulk overlapping peptide approach. Diverse CD4+ T cell responses were observed in most ZIKV-infected participants, while responses to CD8+ T cell epitopes were more limited. Most individuals developed a robust T cell response against epitopes restricted to a single MHC class I supertype and only a single or few CD8+ T cell epitopes overall, suggesting a strong immunodominance phenomenon. Noteworthy is that many epitopes were commonly immunodominant across persons expressing the same class I supertype. Nearly all of the identified epitopes are unique to ZIKV and are not present in Dengue viruses. Collectively, we identified 31 immunogenic peptides restricted by the 6 major class I supertypes and 27 promiscuous class II epitopes. These sequences are highly relevant for design of T cell-targeted ZIKV vaccines and monitoring T cell responses to Zika virus infection and vaccination.

## 1 Introduction

Flaviviruses including Dengue, West Nile, Yellow Fever and Zika are positive sense, single-stranded RNA viruses transmitted by mosquitoes in regions primarily located along the equator. Zika virus (ZIKV) was first identified in a monkey from the Ugandan Zika Forest in 1947, and the first confirmed human case was reported in Uganda in 1963 (1). Few confirmed human cases were found for the next several decades, until an outbreak in 2007 in the Yap Islands resulted in 49 confirmed cases; impressively, it is estimated that 75% of the population became infected during this outbreak (1). Individuals infected with ZIKV were asymptomatic or displayed only mild illness characterized by fever, rash, joint pain and/or conjunctivitis. A larger outbreak occurred in French Polynesia in 2013-14 in which 340 confirmed cases were found, but as many as 49% of the population of ~270,000 was estimated to have been infected based upon serological surveys (2). During this outbreak, a higher incidence of Guillain-Barré Syndrome (GBS) was observed (3). In 2014, autochthonous transmission of ZIKV was detected in Chile (Easter Island) coinciding with transmission in the Pacific Islands, and by 2015, circulation in Brazil was widespread and ZIKV infection was associated with cases of GBS as well as birth defects. Most concerning were associations with severe brain (microcephaly) and eye defects and neurodevelopmental abnormalities in infants born to women with ZIKV infections during pregnancy (1, 4). In one study, 1450 children born to women with laboratory evidence of ZIKV infection during pregnancy were evaluated: 203 (14%) had a ZIKV-associated neurologic abnormality or birth defect, and 84 (6%) had microcephaly (5). Though much has been learned in the past several years, the exact causes of ZIKV infection-induced neurologic complications during fetal development and adulthood have yet to be elucidated.

There are currently no FDA-approved treatments for ZIKV infection or preventive vaccines. ZIKV infection induces rapid and durable neutralizing antibody and T cell responses in humans. In an observational longitudinal study of ZIKV-infected persons, we reported that ZIKV-specific neutralizing antibody and T cell responses were elicited early during infection in all ZIKV-infected participants and these titers remained elevated throughout the duration of the study year (6). ZIKV neutralizing antibody titers were significantly higher in dengue virus (DENV)-experienced participants compared with DENV-naïve subjects, consistent with the known cross-reactive nature of antibodies induced by other flavivirus infections (7). ZIKV-specific antibodies, including those present in human sera after receiving formalin-inactivated Zika vaccine (ZPIV), can provide protection against ZIKV challenge in animal models (8). Antibody-dependent enhancement (ADE) has been reported during heterologous infections with different flavivirus infections (e.g., DENV) including in animal models of ZIKV infection (9, 10). However, dengue-naïve and dengue-exposed individuals exhibited similar viral loads and cytokine profiles during ZIKV infection (11). Thus, ADE remains an unlikely cause of severe disease during human ZIKV infection based on epidemiologic data. It is worth noting that most Zika vaccine development strategies focus on generation of neutralizing antibody responses.

We now report a detailed study of T cell responses induced by ZIKV infection in the cohorts described above. Flavivirus-specific T cells are induced rapidly in infected individuals and have been shown to mediate protection in animal models (12–14). In fact, CD8+ cells induced by DENV infection protect against ZIKV infection (15, 16) similar to findings from other heterologous flavivirus studies (17, 18). HLA-B7 transgenic mice vaccinated with DENV/ZIKV cross-reactive HLA-B7-restricted epitopes developed CD8+ T cell responses that were protective against ZIKV infection (19). Several ZIKV T cell epitopes have been identified using known DENV epitopes as the starting point for sequence-based comparisons. In the study presented here, we utilized advanced matrix-based MHC-binding prediction algorithms for 6 human class I and 9 human class II supertypes to identify T cell epitopes spanning the entire ZIKV open reading frame.

## 2 Materials and methods

### 2.1 Study design and participant description

Volunteers with suspected ZIKV infection after recent travel to Zika-endemic regions were recruited at US sites between July 2016 and September, 2017 as part of a natural history study. The study design and primary results were published previously (6). ZIKV infection was established by ZIKV PCR positive testing or ZIKV-specific IgM and neutralizing antibody test results indicative of infection (6). It was reported that 68.9% of the recruited Zika-infected donors were female, 28.9% Hispanic (71.1% non-Hispanic) and the median age was 44 (6). In this study, a subset of samples was evaluated from 14 ZIKV-naïve and 41 ZIKV-infected participants collected 1-6 months post-predicted infection. Of the 41 ZIKV-infected participants, 13 were considered flavivirus-experienced (6) based upon high baseline DENV-specific neutralizing antibody titers (≥250). Peripheral blood mononuclear cells (PBMC) were isolated from whole blood using standard techniques, cryopreserved, and stored in vapor phase nitrogen until use. Saliva samples were utilized for HLA typing (ProImmune), and 4-digit resolution typing results were mapped to class I and II supertypes based on work described by Southwood and Sidney et al (20, 21).

### 2.2 Immunoinformatics

Algorithms developed at EpiVax, Inc were used to identify CD4+ and CD8+ T cell epitopes from the reference ZIKV strain PRVABC59 (3,423 amino acids; Genbank protein accession number AMC13911.1). The immunoinformatic algorithms used in this work are available in the Interactive Screening and Protein Reengineering Interface (ISPRI) web-based toolkit, which provides seamless transition of data from silo to silo (22–30). Predicted binding for 9 common class II alleles (DRB1*0101, DRB1*0301, DRB1*0401, DRB1*0701, DRB1*0801, DRB1*0901, DRB1*1101, DRB1*1301, and DRB1*1501) expressed by >95% of the human population was assessed using the matrix-based MHC binding algorithm EpiMatrix (22–24). This algorithm scores individual 9-mer frames for predicted binding to each allele, and assessments with Z-scores above 1.64 were considered potential binders. Though Z-scores are a measure of binding probability rather than binding affinity, in practice higher Z-scores are correlated with both higher rates of binding and higher binding affinity. The binding groove of HLA class I and HLA class II molecules can accommodate, in most cases, 9 amino acids. The binding groove of the class I molecule is “closed-ended”. As a result, these molecules can only bind short peptides, usually ranging from 8 to 11 amino acids in length. The binding groove of the class II molecule is “open-ended” and can accommodate longer peptides. However, the core of the binding groove, the part that controls the interaction between the peptide ligand and the presenting MHC is still limited to just 9 amino acids. Class II ligands are longer, typically 15 to 25 amino acids in length. This is because class II ligands must be stabilized by the interaction of leading and trailing “flanking” residues with the outside of the class II HLA molecule. The modeling tool used at EpiVax, the EpiMatrix system, evaluates all possible 9-mer cores for binding. The Clustimer algorithm finds cores that are overlapped and constructs longer peptides that contain multiple binding cores and adds n- and c-terminal flanking residues in order to construct peptides that are appropriate for synthesis and testing. EpiMatrix was also employed to identify peptides predicted to bind alleles representative of the 6 major class I supertypes (A*0101, A*0201, A*0301, A*2402, B*0702, and B*4403) cumulatively expressed by >95% of humans. The ClustiMer algorithm (25, 26) was used to identify longer sequences (16-27 amino acids) predicted to bind multiple class II alleles, and cluster scores above 10 were further evaluated.

Complete sequences from 104 additional ZIKV strains available in 2016 were utilized to evaluate the conservation of epitopes identified from strain PRVABC59 in diverse clinical isolates (from Africa, Asia and the Americas) using the Conservatrix algorithm (27). JanusMatrix (28–30) is a unique EpiVax tool which was used to investigate potential cross-reactivity between predicted ZIKV epitopes and human proteins as well as between different flaviviruses. This tool is particularly useful in identifying (and triaging) “cross-conserved” epitopes which have human homology at the T cell receptor (TCR) face and are more likely to cause off-target effects (e.g., autoimmunity or regulatory T cell responses). Only peptides with low cross-conservation with human proteins (JanusMatrix homology score below 2) were selected. Selected peptides were synthesized to high purity (>80%) by 21^st^ Century Biochemicals, Inc, suspended to 1 mg/ml (each individual peptide) in dimethyl sulfoxide (DMSO) and frozen at -80°C in single-use aliquots until use. Cross-conservation analyses of confirmed epitopes were performed using a panel of DENV strains by JanusMatrix. Additionally, validated peptide sequences shown to be immunogenic in samples from ZIKV-infected participants expressing the appropriate HLA alleles were evaluated using the Immune Epitope Database (IEDB) (31) to determine the novelty of the identified epitopes.

### 2.3 Zika virus and peptide library

ZIKV strain PRVABC59 (BEI 50240) was propagated in Vero cells (ATCC CCL-81) and quantified using focus forming assays (FFA) as previously described (13, 32). Overlapping peptides (15-mers, overlapping by 10 amino acids) spanning the entire polyprotein of ZIKV strain PRVABC59 (GenBank: AMC13911.1) were synthesized and prepared into pools of 49 peptides each (except the final pool, N, which consisted of 46 peptides) by 21^st^ Century Biochemicals, Inc, as shown in **Table 1**. Peptides were suspended to 1 mg/ml (each individual peptide) in DMSO and stored at -80°C until use.

**Table 1:** Design of overlapping peptide pools spanning Zika viral proteome.

Pool	No. of 15mers in pool	Amino acids	Proteins
A	49	1-255	C, P, M
B	49	246-500	M, E
C	49	491-745	E
D	49	736-990	E, NS1
E	49	981-1235	NS1, NS2A
F	49	1226-1480	NS2A, NS2B
G	49	1471-1725	N2SB, NS3
H	49	1716-1970	NS3
I	49	1961-2215	NS3, NS4A
J	49	2206-2460	NS4A, NS4B
K	49	2451-2705	NS4B, NS5
L	49	2696-2950	NS5
M	49	2941-3195	NS5
N	46	3186-3423	NS5

### 2.4 ELISPOT assays

PBMC were thawed, washed, and suspended in ELISPOT assay medium (RPMI1640 medium supplemented with 10% fetal bovine serum, L-glutamine, penicillin and streptomycin). IFN-γ ELISPOT assays were set up according to manufacturer recommendations (BD Biosciences #551849). Briefly, antibody-coated plates were washed and blocked with ELISPOT medium for at least 2 hours at room temperature. PBMC (3x10^5^/well) were stimulated with DMSO as a negative control (0.1% final concentration matched to peptide condition concentrations), peptide pools (1 µg/ml of each peptide), individual peptides (1 µg/ml), ZIKV (multiplicity of infection = 1), and Phorbol 12-myristate 13-acetate as a positive control (PMA; 5 ng/ml). After 22-24 hours of incubation, ELISPOT assays were developed according to manufacturer recommendations. Spots were visualized using aminoethyl carbazole (AEC) substrate solution, reactions stopped using multiple water rinses, and plates decontaminated by submersion in 2% formaldehyde. ELISPOT plates were scanned using a CTL S6 Universal-V Analyzer (ImmunoSpot 6.0 plate reader; ImmunoCapture Version 6.6) and spots enumerated with ImmunoSpot CTL cell counting software (version 1.3).

### 2.5 Statistics and analysis

Proprietary immunoinformatic tools developed at EpiVax were utilized for selection of putative T cell epitopes and epitope clusters. The numbers of spot-forming cells (SFC) per million PBMC are shown in graphs generated using GraphPad Prism, version 9. Statistical analyses of composite data from different cohorts of participants were evaluated using Wilcoxon rank-sum tests. The threshold for positive responses to a specific peptide were determined by being a) ≥ 10 SFC/million PBMC, b) >2 standard deviations (SD) above the mean of the sample’s DMSO response, and c) > 2 SD above the mean of all samples’ DMSO responses. Hit rates for specific peptides were calculated as the number of participants with a positive response divided by the number of participants tested.

## 3 Results

### 3.1 Immunoinformatic selection of human T cell epitopes expressed by ZIKV

**Figure 1:**
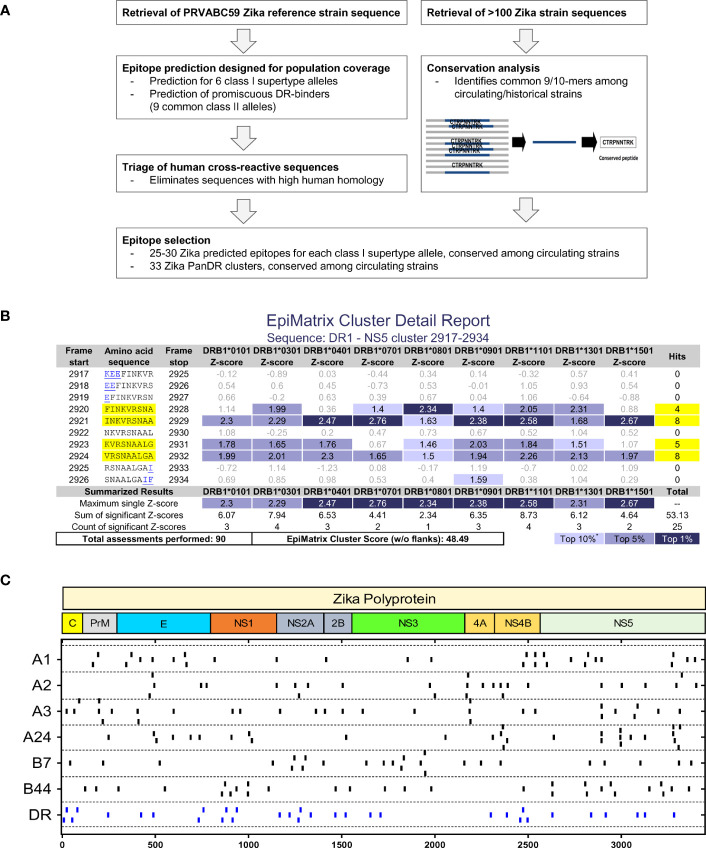
Immunoinformatic strategy to identify MHC I and MHC II T cell epitopes. **(A)** The entire sequence of ZIKV strain PRVABC59 and 104 additional ZIKV strains were parsed into 9- and 10-mer frames, and conserved sequences predicted to bind to 6 MHC I supertypes and 9 common MHC II alleles were identified using Conservatrix and EpiMatrix algorithms. Promiscuous panDR clusters (DR) were generated from overlapping frames predicted to bind multiple MHC II alleles using the ClustiMer algorithm (16-27 amino acids). Sequences with high homology with human proteins (potential human cross-reactive sequences) were excluded using the JanusMatrix algorithm. **(B)** Shown is an example (Cluster DR1) of an EpiMatrix Cluster Report. Z-score indicates the potential of a 9-mer frame to bind to a given HLA allele; the strength of the score is indicated by the blue shading. Scores in the top 5% (Z-score ≥1.64) are considered “Hits”. *Z-scores in the top 10% are considered elevated, other scores grayed for simplicity. Frames containing four or more alleles scoring above 1.64 are referred to as EpiBars and are highlighted in yellow. Flanking amino acids, added to stabilize the cluster during *in vitro* testing, are underlined. The EpiMatrix Cluster Score is derived from the sum of Z-scores of EpiMatrix hits, normalized for the length of the cluster. Thus, EpiMatrix Cluster Score represents the excess or shortfall in predicted aggregate immunogenicity relative to a random peptide standard. Cluster Scores above 10 indicate significant potential for promiscuous response. **(C)** The 25-30 conserved ZIKV peptides predicted to bind the 6 MHC I supertypes and 33 clusters predicted to promiscuously bind human HLA-DR were synthesized for further study. The locations of each putative epitope or cluster within the ZIKV polyprotein are shown.

Two strategies employing IFN-γ ELISPOT assays were developed to evaluate T cell responses and their specificities (molecular targets) induced by ZIKV infection: targeted immunoinformatics *vs*. unbiased whole genome approaches. The overall strategy to identify putative T cell epitopes is shown in **Figure 1A**. The full-length polyprotein sequence from ZIKV strain PRVABC59 was parsed into 9-mer frames and scored for predicted binding to 9 common class II DRB1 alleles cumulatively expressed by 95% of the population using the previously described and validated EpiMatrix algorithm (33). For each allele, frames scoring >1.64 on the EpiMatrix Z-scale are determined as likely MHC binders. In parallel, the Conservatrix algorithm was used to evaluate the conservation of each scored 9-mer frame within a panel of 104 ZIKV isolates. The ClustiMer tool was used to identify overlapping 9-mer frames that contain predicted class II epitopes, and generated T cell clusters which are longer, epitope dense sequences with broad promiscuity (25, 26). These T cell clusters were examined for conservation with human proteins utilizing the JanusMatrix algorithm which can identify sequences predicted to bind HLA and have similar residues interacting with the TCR face. Clusters with high homology (JanusMatrix >2.0) to human proteins overall and at the TCR face were identified and excluded from further study, to avoid identification of epitopes with potential to induce regulatory/suppressive T cells or autoimmunity (28–30). The top clusters selected for synthesis and *in vitro* testing ranged from 16-27 amino acids in length, contained multiple predicted MHC ligands (combined Z-scores of 10.9 – 48.5; average of 25.7), and were highly conserved in the panel of 104 ZIKV isolates (95% on average at ≥80% sequence identity). An example of an EpiMatrix Cluster Report for Cluster DR1 is shown (**Figure 1B**) with the locations of the selected promiscuous DR clusters (DR) in the ZIKV genome (**Figure 1C**). A similar approach was utilized to identify putative CD8+ T cell targets. The ZIKV polyprotein sequence was parsed into 9- and 10-mer frames, and predicted binding to each of 6 HLA alleles representative of major class I supertypes (A1, A2, A3, A24, B7 and B44) was performed using EpiMatrix as described above. Selected epitopes on average were conserved at ≥80% amino acid homology in >95% of the 104 strains and were present throughout the length of the entire ZIKV polyprotein (**Figure 1C**).

### 3.2 Evaluation of T cell responses in volunteers previously infected with ZIKV

**Figure 2:**
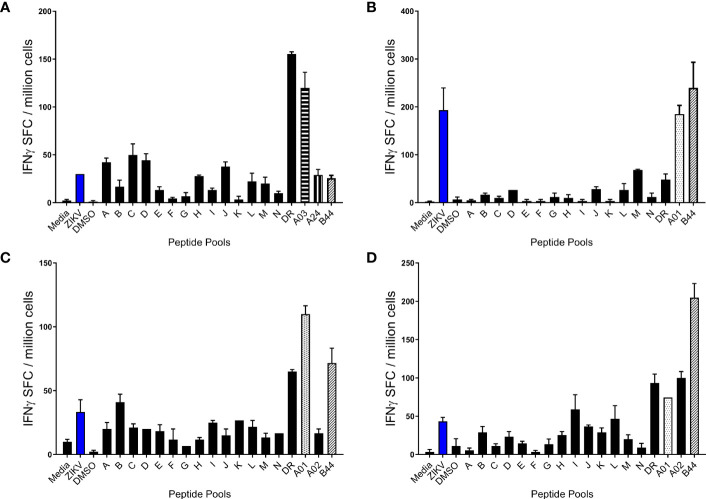
Human T cell responses induced by ZIKV infection. PBMC from four different participants **(A–D)** previously infected with ZIKV were studied in overnight IFN-γ ELISPOT assays. Each sample was stimulated with live ZIKV or media control, overlapping peptide pools A-N, predicted promiscuous panDR clusters (DR), or pools of ZIKV peptides predicted to bind MHC I supertypes. While all sets of PBMC were stimulated with live ZIKV, overlapping peptide pools, and the MHC II pool (DR), participant PBMC were only stimulated with MHC I peptide pools matched to the participant HLA type. Shown are the means + SE of the numbers of IFN-γ spot forming cells (SFC) per million PBMC from 4 different Zika-infected participants.

PBMCs from ZIKV^pos^ and ZIKV^neg^ participants were stimulated in overnight IFN-γ ELISPOT assays with live ZIKV, pools of overlapping ZIKV peptides (A-N), and pools of peptides predicted to bind promiscuously to HLA-DR alleles, or to each of the 6 MHC class I supertypes. PBMCs were only stimulated with peptide pools matching their HLA class I typing results (e.g.,HLA A1+, A2+, B7+, B44+ PBMCs stimulated with A1, A2, B7, and B44 peptide pools). Shown in **Figure 2** are results from four different ZIKV^pos^ volunteers. All four of the volunteers responded to the promiscuous panDR clusters (DR) and at least one pool predicted to bind to one of the volunteer’s MHC I supertypes. Responses to live ZIKV and the overlapping 15-mer peptide pools were varied, and T cell responses to the overlapping peptides were lower, on average, than the maximal responses induced by the pools predicted using immunoinformatic tools.

**Figure 3:**
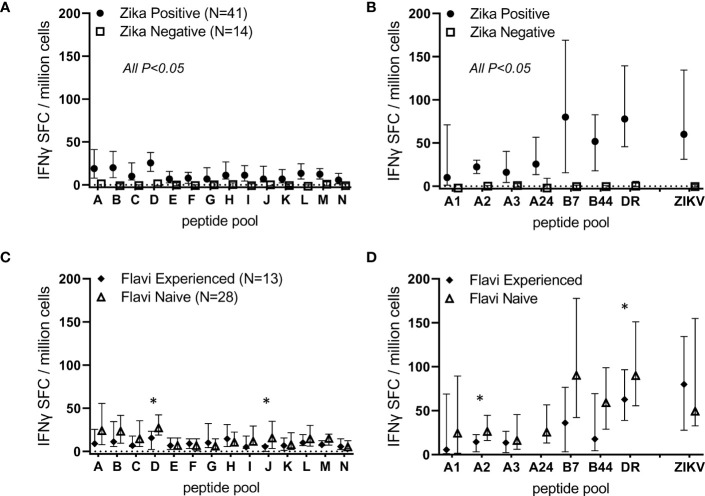
ZIKV infection induces T cell responses directed against overlapping peptides spanning the viral proteome. PBMC from 14 ZIKV-uninfected and 41 Zika-infected participants were studied in overnight IFN-γ ELISPOT assays. Each sample was stimulated with live ZIKV (or media control), overlapping peptide pools A-N, predicted promiscuous panDR clusters (DR), or pools of ZIKV peptides predicted to bind MHC I supertypes. Participant PBMC were only stimulated with MHC I peptide pools matched to the participant HLA type. Shown are DMSO subtracted medians and 25^th^/75^th^ percentiles. Statistical significance was determined *via* Wilcoxon rank-sum test. In **(A, B)**, all comparisons between Zika negative and Zika positive participants were significant (P<0.05). In **(C, D)**, responses in ZIKV-positive participants were stratified based on dengue serology and classified as Flavivirus naïve (N=28) or experienced (N=13). Overlapping peptide pools D and J and predicted pools A2 and DR were determined to be significantly different between previously flavivirus experienced and flavivirus naïve Zika participants (*P<0.05 by Wilcoxon rank-sum tests).

These observations are further demonstrated across the cohort of 41 ZIKV^pos^ participants (**Figures 3A, B**). A total of 14 participants determined to be ZIKV^neg^ (uninfected) were also studied in these assays, and very little background was observed in these individuals. Statistically significant differences were observed between ZIKV positive and negative participants for all the tested overlapping and predicted peptide pools, as well as for live ZIKV (**Figures 3A, B**). We next determined whether live ZIKV and ZIKV peptide-specific T cell responses were different in Zika-infected, but previously flavivirus-naïve participants, versus responses from previously flavivirus-experienced, Zika-infected participants (determined by dengue serology). Shown in **Figures 3C, D** are results from ZIKV^pos^ participants segregated by previous flavivirus experience. Responses to overlapping and predicted ZIKV peptide pools were numerically higher in ZIKV^pos^ but previously flavivirus naïve subjects than in ZIKV^pos^ and flavivirus-experienced participants, with some response differences achieving statistical significance (overlapping pools D and J, and HLA-A2- and panDR-predicted pools).

### 3.3 Identification of individual epitopes eliciting responses in PBMCs from ZIKV-infected participants

**Figure 4:**
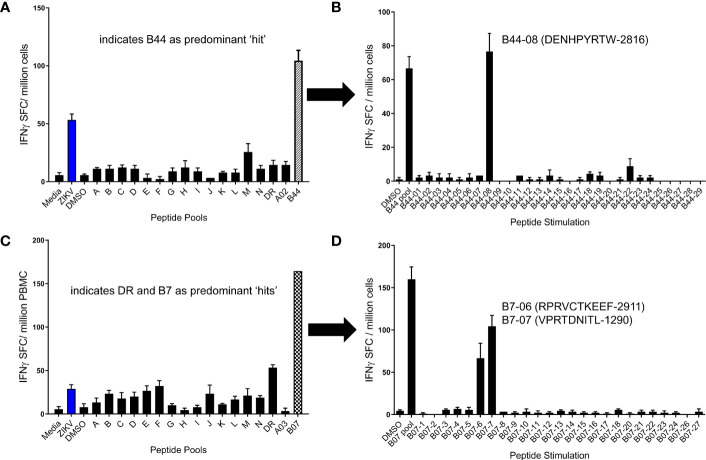
Identification of immunogenic ZIKV peptides using a 2-step ELISPOT assay. PBMC from 2 different participants previously infected with ZIKV were studied in overnight IFN-γ ELISPOT assays. Shown in **(A, C)** are screening assay results from two individuals in which the predominant peptide pool responses were to B44 **(A)** and B07 **(C)**, respectively. In follow up assays, participant PBMC were stimulated with individual peptides from these pools to identify individual epitopes inducing responses. The entirety of the B44 peptide pool response for the participant tested in **(A, B)** was directed to a single peptide: B44-08 (ZIKV2816; **B**). The B7 peptide pool response in **(C, D)** were directed against two tested peptides, B7-06 (ZIKV2911) and B7-07 (ZIKV1290).

After determining the peptide pools which induced positive responses in each volunteer, we next sought to identify the individual peptides responsible for the positive responses. For example, shown in **Figure 4** are peptide pool screening assay results from participants that showed responses to the B44 pool (A) or the B7 pool (C). In the follow-up ELISPOT assays, PBMCs from these same volunteers were stimulated with the same peptide pool (e.g., B44 and B7 pools) as well as with each of the individual peptides. As shown in panel B, the majority of T cells from the volunteer responding to the B44 peptide pool were directed to a single peptide: B44-08 (NS5_2816_ DENHPYRTW). T cells from the other volunteer responsive to the B7 peptide pool responded to only two individual peptides contained within the B7 pool (panel D; B7-06: NS5_2911_ RPRVCTKEEF and B07-07: NS2A_1290_VPRTDNITL).

**Figure 5:**
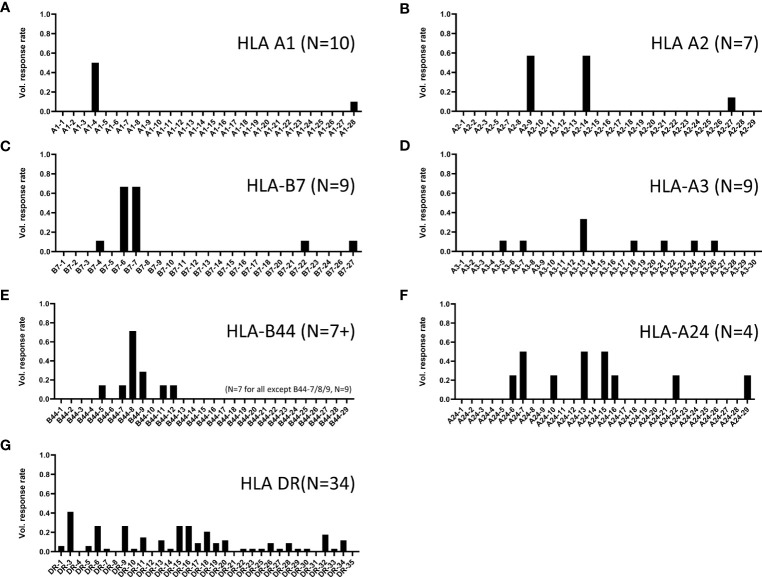
ZIKV participant response rates to predicted MHC I and II binders. Shown are the positive response rates of ZIKV-participant PBMC studied in IFN-γ ELISPOT assays with individual peptide stimulations (x-axis). Shown are responses to peptides predicted to bind **(A)** HLA-A1, **(B)** HLA-A2, **(C)** HLA-B7, **(D)** HLA-A3, **(E)** HLA-B44, **(F)** HLA-A24, and **(G)** multiple MHC II alleles (pan HLA-DR). Response rate calculated as number of tested participants with positive response ÷ number of tested participants. The number of participants tested for each set are shown in parenthesis on each panel.

Similar assays were conducted with additional volunteers, and positive responses were determined based on calculations described in *Methods*. Shown in **Figure 5** are the positive response rates of volunteers tested for each class I supertype (panels A-F) and promiscuous class II cluster sets (panel G). Only 2 epitopes were identified using PBMCs from 10 participants expressing HLA-A1 (panel A). In contrast, 8 individual HLA-A24-restricted T cell epitopes were identified (panel F), using PBMCs from just 4 participants expressing HLA-A24 supertype alleles. Diverse responses to the class II panDR clusters were observed, with 27 unique peptides inducing responses in at least 1 of the 34 participant PBMCs tested. Many class I supertype responses in different volunteers focused on a single or select few of the predicted epitopes. In addition, we found many examples where at least 50% of volunteers expressing alleles within a given supertype responded to the same immunodominant epitopes. In contrast, response rates to the different DR clusters were in general more diverse, with lower frequencies of responses to individual epitopes than those observed for class I. In total, 31 MHC I-restricted T cell epitopes and 27 MHC II-restricted epitope clusters were identified, as summarized in **Tables 2**–**4**. The identified epitopes are located throughout the ZIKV proteome in both structural and non-structural gene products and conserved throughout various ZIKV isolates. It is worth noting that many of the identified epitopes have been reported previously, and nearly all of the T cell epitopes identified using our strategy are ZIKV-specific as they are not conserved in other flaviviruses, including DENV 1-4 isolates (**Supplemental Table 1**).

**Table 2:** Validated class I supertype-restricted ZIKV epitopes.

Peptide Name	Sequence	ZIKV Protein	Position	A0101 Z-score	A0201 Z-score	A0301 Z-score	A2402 Z-score	B0702 Z-score	B4403 Z-score	ZIKV conservation at 80% identity (104)
A1-04	GLDFSDLYY	E	485	4.09	1.02	2.19	-1.14	-1.2	0.24	98
A1-28	ETACLAKSY	NS5	3272	3.04	-0.72	0.94	-0.1	-0.39	1.61	100
A2-09	YLQDGLIASL	NS3	2000	1.31	3.12	0.27	0.88	1.01	0.07	100
A2-14	ALTTFITPAV	NS4B	2314	-0.19	2.91	1.17	-0.06	0.71	0.75	99
A2-27	MPFYAWDFGV	NS4B	2353	-0.97	2.16	0.8	1	3.2	0.13	99
A03-5	MSALEFYSYK	NS5	2539	2.16	0.56	3.51	0.47	-0.44	0.49	98
A3-07	VVYGTCHHK	PrM	198	1.07	1.39	3.42	0.41	-0.44	-1.71	102
A3-13	RVSPFGGLK	ancC	23	1.69	0.27	3.28	0.23	0.78	-0.53	72
A3-18	VTLPSHSTRK	PrM	217	1.95	-0.19	3.2	-0.37	-0.66	-0.29	96
A3-21	STLWEGSPNK	NS4B	2473	0.66	0.56	3.16	-0.68	0.1	-0.27	100
A3-24	WVVYGTCHHK	PrM	197	1.3	0.51	3.08	0.86	-0.76	-0.59	102
A3-26	VTCAKFACSK	E	404	0.79	0.66	3.02	0.06	-0.25	-0.86	95
A24-06	TYTDRRWCF	NS3	2057	1.19	-0.48	-0.11	4.03	1.44	1.92	100
A24-07	HYMYLIPGL	NS4B	2388	-0.44	1.57	-0.16	3.95	0.51	1.15	97
A24-10	EWFHDIPLPW	E	506	-0.96	-0.43	-0.44	3.91	-1.33	1.63	98
A24-13	EWPKSHTLW	NS1	1018	0.17	-1.64	-1.18	3.74	-1.71	1.82	100
A24-15	DWVPTGRTTW	NS5	3308	-0.32	-1.05	-0.91	3.57	-0.7	1.25	101
A24-16	SYSLCTAAF	E	594	0.59	-0.12	-0.46	3.55	1.78	1.24	100
A24-22	TWSIHGKGEW	NS5	3316	0.48	-1.04	-0.57	3.17	-0.24	0.05	100
A24-29	LMIGCYSQL	NS4B	2365	0.58	2.86	1.17	2.75	1.41	1	103
B7-04	APTRVVAAEM	NS3	1725	-1.36	-0.53	-1.38	-0.83	3.79	0.82	102
B7-06	RPRVCTKEEF	NS5	2911	-0.72	-1.3	-0.27	2.56	3.7	-0.11	101
B7-07	VPRTDNITL	NS2A	1290	-1.19	0.5	-0.5	0.1	3.64	0.77	100
B7-22	RPRKEPESNL	NS1	1130	-0.93	-0.87	-0.26	0.89	3.29	0.08	99
B7-27	MPFYAWDFGV	NS4B	2353	-0.97	2.16	0.8	1	3.2	0.13	99
B44-05	SEHAETWFF	NS5	2807	0.32	0.24	0.24	2.02	0.12	4.15	89
B44-07	MENIMWRSV	NS1	857	0.61	0.49	0.02	0.26	1.27	4.04	101
B44-08	DENHPYRTW	NS5	2816	-0.06	-2.1	-1.24	0.99	-1.07	3.95	102
B44-09	LEMQDLWLL	NS5	3151	0.32	1.7	-0.37	2.12	0.81	3.81	97
B44-11	REDLWCGSLI	NS5	3364	0.62	1.02	1.01	1.46	0.92	3.69	101
B44-12	GECQSCVYNM	NS5	2966	-1.57	-0.19	-0.13	0.84	0.97	3.67	100

Z-scores indicate the potential of a specific peptide to bind to an MHC molecule. Z-scores in the top 5% (≥1.64) are shaded in blue and considered high potential MHC binders. Top 1% Z-scores (≥2.32) are even more likely to bind MHC, whereas Z-scores in the top 10% (>1.28, <1.64) are not statistically significant and considered “near misses” capable of binding at generally lower frequency and affinity. ZIKV Conservation shown as number of the 104 ZIKV isolate sequences with 80% identity to predicted sequence from reference Zika virus strain PRVABC59.

**Table 3:** Validated ZIKV promiscuous MHC II clusters.

Peptide Name	Source Protein	Cluster Address	Cluster Sequences	EpiMatrix Cluster Score	ZIKV conservation at 80% identity (104)
DR1	NS5	2917 - 2934	KEE**FINKVRSNAALGA**IF	48.49	100
DR3	ancC	0081 - 0106	IKK**FKKDLAAMLRIINARKEKKR**RGA	36.62	97
DR5	NS2A	1279 - 1296	**ALAWLAIRAMVVPRT**DNI	22.54	100
DR6	E	0423 - 0444	ENLE**YRIMLSVHGSQHSGM**IVN	35.92	100
DR7	NS5	3128 - 3153	TYA**LNTFTNLVVQLIRNMEAEEV**LEM	34.77	96
DR9	ancC	0008 - 0028	SGG**FRIVNMLKRGVARVS**PFG	33.48	92
DR10	NS3	1521 - 1543	TDG**VYRVMTRRLLGSTQVGV**GVM	32.91	100
DR11	NS2A	1167 - 1191	QEG**LKKRMTTKIIISTSMAVLV**AMI	32.83	101
DR13	NS5	2838 - 2857	ASS**LINGVVRLLSKPWD**VVT	29.35	101
DR14	NS2A	1220 - 1242	VAH**LALIAAFKVRPALLVSF**IFR	28.73	103
DR15	ancC	0024 - 0041	VSP**FGGLKRLPAGLL**LGH	28.59	97
DR16	NS5	3283 - 3305	MWQ**LLYFHRRDLRLMANAIC**SSV	27.75	101
DR17	NS4B	2300 - 2323	DID**LRPASAWAIYAALTTFIT**PAV	26.44	99
DR18	NS5	3087 - 3113	LAI**IKYTYQNKVVKVLRPAEKGKT**VMD	26.34	97
DR19	ancC	0053 - 0070	FLR**FTAIKPSLGLIN**RWG	25.61	99
DR20	E	0489 - 0505	SDL**YYLTMNNKHWL**VHK	25.38	98
DR22	NS2A	1334 - 1354	KGS**VKKNLPFVMALGLTA**VRL	23.41	98
DR23	prM	0246 - 0268	RVEN**WIFRNPGFALAAAAIA**WLL	22.64	95
DR25	E	0758 - 0776	IGT**LLMWLGLNTKNGS**ISL	20.1	101
DR26	NS4B	2473 - 2495	KSTL**WEGSPNKYWNSSTATSL**CNI	20.1	98
DR27	NS3	1654 - 1674	NGV**VIKNGSYVSAITQGR**REE	20.08	98
DR28	NS4B	2497 - 2519	RGS**YLAGASLIYTVTRNAGL**VKR	19.92	99
DR29	NS5	2631 - 2654	EEPV**LVQSYGWNIVRLKSGVD**VFH	19.12	97
DR30	NS3	1708 - 1733	PEI**VREAIKTRLRTVILAPTRVV**AAE	19.05	101
DR32	NS2B	1466 - 1481	MRE**IILKVVLMTI**CGM	11.92	101
DR33	NS1	0859 - 0876	NIM**WRSVEGELNAIL**EEN	11.57	101
DR34	NS4	0937 - 0955	KPL**KHRAWNSFLVEDH**GF	10.94	98

Cluster scores indicate the potential of a specific peptide to bind to a set of 9 common human MHC II molecules. Higher Cluster scores suggest greater binding promiscuity; scores >10 are considered significant. The cluster core within the cluster sequence is indicated by bold font, while the n- and c- terminal flanking residues are shown in regular font. ZIKV Conservation shown as number of the 104 ZIKV isolate sequences with 80% identity to predicted sequence from the reference Zika virus strain PRVABC59.

**Table 4:** Summary of ZIKV epitopes identified in this study.

MHC	Peptide Set or Supertype	PBMC Sets Studied	Positive Responses	Epitopes Inducing Positive Response
Class II				
	PanDR Clusters	34	106	27
Class I				
	A1	10	6	2
	A2	7	9	3
	A3	9	9	7
	A24	4	11	8
	B7	9	15	5
	B44	7 - 8	11	6
			167	58

## 4 Discussion

Human B cell and antibody responses to ZIKV infection have been well characterized by multiple groups, including ours (6, 34–36), and over the past few years much has been learned about ZIKV-specific T cells (6, 37–41). In our current work, we corroborate findings from other studies demonstrating strong ZIKV-specific effector T cell responses after ZIKV infection (**Figure 3**). In addition to identifying potent T cell responses to live ZIKV stimulation, we also found heterogenous T cell responses to overlapping peptide pools and pools of peptides predicted to bind class I supertypes and common class II HLA molecules (**Figures 2**, **3**). These responses are consistent with those reported elsewhere in which ZIKV-infected participant T cells responded to ZIKV peptide megapools containing multiple overlapping (or predicted) ZIKV peptides (42, 43). Using these immunoinformatically predicted peptides and a two-step ELISPOT method, we identified and validated 58 T cell epitopes (31 MHC I-restricted CD8+ T cell epitopes, and 27 promiscuous MHC II-restricted CD4+ T cell epitope clusters, summarized in **Tables 2**–**4**) which warrant further study as potential components of novel T cell-targeted ZIKV vaccines.

The T cell studies presented here extend the data previously published using samples from the same cohort of Zika-infected participants (6). Both our initially published data and the work reported here showed that ZIKV-specific CD4+ and CD8+ T cell responses were targeted to sequences across the ZIKV polyprotein. Also, in both reports, T cell responses in acutely ZIKV-infected persons were higher in previously flavivirus-naïve participants than in previously flavivirus-experienced subjects. It is possible that acute ZIKV infection results in higher T cell responses in flavivirus-naïve individuals because these persons develop higher viral loads in the absence of pre-existing cross-reactive neutralizing antibody responses. This hypothesis is consistent with previous reports indicating that despite significant cross-reactivity between ZIKV and Dengue comparing B cell epitopes, very little cross-reactivity between ZIKV and other flavivirus has been found studying T cell epitopes (42). Our results presented here do provide distinct and novel information compared with our earlier publication in that: 1) the assays used were different (IFN-γ ELISPOT versus direct *ex vivo* intracellular cytokine staining), 2) the cytokines used to detect positive responses were different (IFN-γ alone associated with protective effector functions versus at least one of three cytokines measured by ICS including IFN-γ, IL-2 or TNF, which may detect less differentiated T cells which may not contribute to optimal protective effects), and 3) we focus here on more broadly relevant T cell epitopes across HLA supertypes that might provide better population level coverage if included in future T cell-targeted vaccines.

In general, it appears that peptide pools generated using advanced algorithms out-performed overlapping peptide approaches (**Figure 3**). Median magnitudes of T cell responses to pools of predicted epitopes were up to four times higher than responses to overlapping peptide pools. This suggests that the immunoinformatic prediction-based strategy may be more useful for identification of relevant epitopes. While all 9-10-mer class I epitopes would be represented in at least one peptide in the 15-mer overlapping peptide pools, it is possible that some class II peptides would not be ideal for binding to class II molecules (due to truncated or off-center binding motifs and/or missing flanking positions). The immunoinformatic predictions included epitopes shown to induce responses shared by up to 60% of all volunteers tested expressing the same class I supertype. This may be due to the inherent focus of our prediction strategy which specifically targets identification of broadly relevant T cell epitopes across all individuals sharing the same supertype. The described tools predict HLA binding potential for a panel of 9 common class II HLA alleles. These alleles were selected because they are common within the human population worldwide and relatively distinct from each other. EpiVax refers to these alleles as supertypes. Each supertype is functionally equivalent or nearly equivalent to many additional family member alleles (i.e., alleles within a supertype family share a set of common peptide binding preferences). Southwood, Sette and Sidney first described the supertype phenomenon for both class I and class II HLA (21, 44, 45). Taken collectively, the 9 class II supertype alleles, along with their respective family members, cover well over 95% of HLA types present in most human population groups. Similar population coverage was determined for class I. Epitopes were identified based on predicted binding to a panel of six common class I alleles: A*0101, A*0201, A*0301, A*2402, B*0702, and B*4403. These alleles are supertypes. Each one is functionally equivalent to or nearly equivalent to many additional family member alleles. Taken collectively, these 6 supertype alleles, along with their respective family members, cover well over 98% of the human population, and some (such as HLA-A02) are expressed by nearly half of the human population. T cell epitopes identified using an overlapping approach may be restricted by rare human class I individual alleles and not broadly across all alleles of a supertype and would thus not be as relevant for development of vaccines for protection of diverse populations. Similarly, the panDR T cell epitope clusters studied here are likely to be broadly relevant, as most of the T cell clusters studied induced responses in multiple volunteers. We note that both strategies (overlapping *vs*. predictions) have been utilized to successfully identify immunogenic epitopes (46–49). In fact, we were able to identify the same immunodominant T cell epitope using a single participant’s PBMC with both the overlapping peptide/matrix-based deconvolution approach (VAHYMYLIPGLQAAA) and the immunoinformatic predictive strategy described here (A24-7: HYMYLIPGL) (data not shown). However, our direct comparisons in this study demonstrate that the predictive algorithms may outperform overlapping peptide pools specifically for identifying more epitopes broadly relevant across the population.

Several assay methods are used by investigators to identify human MHC-restricted T cell epitopes. Direct *ex vivo* assays using human PBMCs, such as the ones utilized in this study, identify medium to high frequency effector T cells, and may favor the detection of immunodominant T cell responses over subdominant responses. *In vitro* assays including measurements of T cell expansion after activation (e.g., cultured ELISPOT assays) allow for detection of T cell responses capable of both proliferation and effector functions including central memory T cells, which after activation and proliferation differentiate into effector T cells. Thus, these expanded ELISPOT assays generally identify higher numbers of T cell epitopes (50, 51). However, it is unknown whether direct *ex vivo* or expanded T cell assays are better at predicting T cell responses capable of providing long-term protective immunity. In fact, one of the limitations of the current study is that most of the epitopes identified in our work have not been confirmed to be involved in protective immunity. Many investigators utilize HLA transgenic mice for identification of T cell epitopes, which also offer the opportunity to evaluate *in vivo* protective immunity for certain pathogens (19, 49, 52). Some of the epitopes identified here have been previously reported to be targets of T cells in humans and/or transgenic mice expressing human MHC. For example, ZIKV peptides B7-4, B7-6, and B7-7 identified here (**Figure 5C**) have been shown to be immunogenic after ZIKV infection in HLA-B7 transgenic mice (19). Furthermore, peptide-based vaccines which included two of these epitopes were shown to provide some protective immunity (significant reduction of viremia) against live ZIKV challenge in HLA-B7 transgenic animals (19). The additional epitopes identified in our study could allow development of protective ZIKV vaccines for a higher proportion of humans expressing highly diverse MHC class I and II molecules.

Our results demonstrate different patterns of class I immunodominance at the supertype level, with different volunteers expressing the same supertypes having a different hierarchy of responses to individual supertype peptides (**Figure 2**). However, we also observed high frequency immunodominance at the population level across participants expressing certain supertypes when identifying individual epitopes from immunogenic MHC I supertype peptide pools (**Figure 5**). For example, in 9 participants tested for responses to individual B7 peptides, only 5 epitopes induced a response in at least a single participant (**Figure 5C**). However, PBMC from 6 of 9 HLA-B7-expressing participants responded to 2 of the B07 peptides (B7-06: NS5_2911_ RPRVCTKEEF and B7-07: NS2A_1290_ VPRTDNITL). Similar immunodominance patterns were identified for additional class I supertypes including HLA-A1, -A2, and -B44 (**Figures 5A, B, E**). It is not known whether this immunodominance is due to different levels of antigen expression, specific fragment proteolysis and processing, or more avid binding of the peptides to MHC or TCR. Additional studies are needed to determine the reasons for such immunodominance.

Responses to the promiscuous ZIKV panDR clusters were more diverse (**Figure 5F**) than those observed for class I. However, this was expected because each of the ZIKV clusters were predicted to contain epitopes restricted by multiple commonly expressed HLA-DR molecules (ZIKV clusters were identified for promiscuous binding to 9 common HLA-DR supertype alleles). Some of these clusters induced responses in only one or two individuals (of 34 tested), while several induced responses in >25% of participants (e.g., cluster DR3 with a >40% response rate). In total, T cell responses to 27 of the tested 33 ZIKV DR clusters were detected in at least a single volunteer. Eleven ZIKV DR clusters induced responses in >10% of the tested participants. These clusters are highly relevant for development of T cell-targeted vaccines for use in genetically diverse human populations.

Multiple ZIKV vaccine candidates are in various stages of pre-clinical and clinical development and most of these are designed to generate humoral immunity. Vaccine trials using purified formalin-inactivated ZIKV (ZPIV and TAK-426) have demonstrated induction of ZIKV neutralizing antibody responses (8, 53), though durability and magnitude of responses were found to be suboptimal in at least one report (54). Additional vaccine strategies including DNA (55, 56) and adenoviral vectors (57) expressing preM-ENV have also been evaluated in clinical trials and were observed to also induce anti-ZIKV antibody responses. The majority of T cell epitopes identified in this study are present in proteins not expressed in the most advanced recombinant vaccines studied in humans, since our methods allowed identification of epitopes from both structural and non-structural proteins. Our strategy of identifying immunodominant T cell epitopes should provide critical information towards the development of T cell-targeted ZIKV vaccines that invoke immunity in populations expressing diverse HLA. Future studies should be conducted to engineer multi-epitope vaccines expressing these ZIKV-conserved T cell epitopes devoid of homology to human proteins.

## Data availability statement

The original contributions presented in the study are included in the article/**Supplementary Material**. Further inquiries can be directed to the corresponding author.

## Ethics statement

The studies involving humans were approved by Saint Louis University Institutional Review Board. The studies were conducted in accordance with the local legislation and institutional requirements. The participants provided their written informed consent to participate in this study.

## Author contributions

DH conceptualized the project. CE, KM, CC, AB, FT, and AG conducted experiments, HS, NR, MM, MA, and KT provided human clinical samples. JB, AP and ET contributed reagents. DH, CE, KM, LM, FT, AG, ADG, and CF analyzed data and/or interpreted results. CE and DH wrote the first draft of the manuscript, and all co-authors provided edits and approved the submission of this manuscript. All authors contributed to the article and approved the submitted version.
