# Supplementary material for: Identification of immunodominant T cell epitopes induced by natural Zika virus infection
Source: Front Immunol. 2023 Aug 29;14:1247876. doi: 10.3389/fimmu.2023.1247876 (PMC10497216; doi:10.3389/fimmu.2023.1247876)
Supplement: Supplementary file 1 [file Table_1.pdf]

MHC	Peptide Name	Source Protein	AA Sequence	Human JMX Homology Score	Zika JMX Homology Score (104 sequences)	Denv1 JMX Homology Score (15 sequences)	Denv2 JMX Homology Score (15 sequences)	Denv3 JMX Homology Score (15 sequences)	Denv4 JMX Homology Score (15 sequences)	Positeve entries in IEDB (Homology at 80% similarity)	Negative entries in IEDB (Homology at 80% similarity)
class I	A1-04	E	GLDFSDLYY	0.50	98	0	0	0	0	12	6
class I	A1-28	NS5	ETACLAKSY	0.00	100	0	0	0	0	16	2
class I	A2-09	NS3	YLQDGLIASL	0.00	100	0	0	0	0	7	1
class I	A2-14	NS4B	ALTTFITPAV	0.00	72	0	0	0	0	3	0
class I	A2-27	NS4B	MPFYAWDFGV	0.00	92	0	0	0	0	1	0
class I	A24-06	NS3	TYTDRRWCF	0.50	101	0	0	0	0	14	10
class I	A24-07	NS4B	HYMYLIPGL	0.00	97	0	0	0	0	7	0
class I	A24-10	E	EWFHDIPLPW	0.00	98	0	0	0	0	6	2
class I	A24-13	NS1	EWPKSHTLW	0.00	99	0	0	0	0	4	2
class I	A24-15	NS5	DWVPTGRTTW	0.00	101	0	0	0	0	7	2
class I	A24-16	E	SYSLCTAAF	0.50	95	0	0	0	0	10	0
class I	A24-22	NS5	TWSIHGKGEW	0.00	102	0	0	0	0	3	2
class I	A24-29	NS4B	LMIGCYSQL	0.50	103	0	0	0	0	5	3
class I	A3-05	NS5	MSALEFYSYK	0.00	98	0	0	0	0	1	0
class I	A3-07	PrM	VVYGTCHHK	0.00	100	0	0	0	0	0	0
class I	A3-13	ancC	RVSPFGGLK	1.00	72	0	0	0	0	8	2
class I	A3-18	PrM	VTLPSHSTRK	0.00	96	0	0	0	0	1	0
class I	A3-21	NS4B	STLWEGSPNK	0.00	100	0	0	0	0	5	3
class I	A3-24	PrM	WVVYGTCHHK	0.00	100	0	0	0	0	0	0
class I	A3-26	E	VTCAKFACSK	0.00	70	0	0	0	0	5	1
class I	B44-05	NS5	SEHAETWFF	0.00	96	0	0	0	0	0	1
class I	B44-07	NS1	MENIMWRSV	0.00	73	0	0	0	0	4	0
class I	B44-08	NS5	DENHPYRTW	0.00	102	0	0	0	0	10	6
class I	B44-09	NS5	LEMQDLWLL	0.33	88	0	0	0	0	3	0
class I	B44-11	NS5	REDLWCGSLI	0.00	101	0	0	0	15	8	2
class I	B44-12	NS5	GECQSCVYNM	0.00	73	0	0	0	0	4	1
class I	B7-04	NS3	APTRVVAAEM	0.00	102	0	15	15	15	19	4
class I	B7-06	NS5	RPRVCTKEEF	0.00	101	0	0	0	0	18	4
class I	B7-07	NS2A	VPRTDNITL	0.00	74	0	0	0	0	11	1
class I	B7-22	NS1	RPRKEPESNL	0.00	102	0	0	0	0	9	1
class I	B7-27	NS4B	MPFYAWDFGV	0.00	92	0	0	0	0	1	0
class II	DR1	NS5	KEEFINKVRSNAALGAIF	1.28	100.0	7.88	7.88	0.00	0.00	15	8
class II	DR3	ancC	IKKFKKDLAAMLRIINARKEKKRRGA	1.59	92.59	0.00	0.00	0.00	0.00	3	0
class II	DR5	NS2A	ALAWLAIRAMVVPRTDNI	0.58	71.65	0.00	0.00	0.00	0.00	2	1
class II	DR6	E	ENLEYRIMLSVHGSQHSGMIVN	0.63	99.68	0.00	0.00	0.00	0.00	3	5
class II	DR7	NS5	TYALNTFTNLVVQLIRNMEAEEVLEM	0.36	93.77	0.00	0.00	0.00	0.68	0	1
class II	DR9	ancC	SGGFRIVNMLKRGVARVSPFG	1.70	89.65	0.00	0.00	0.00	0.00	4	3
class II	DR10	NS3	TDGVYRVMTRRLLGSTQVGVGVM	1.05	100.0	0.00	0.00	0.00	0.00	0	3
class II	DR11	NS2A	QEGLKKRMTTKIIISTSMAVLVAMI	0.55	89.95	0.00	0.00	0.00	0.00	0	2
class II	DR13	NS5	ASSLINGVVRLLSKPWDVVT	1.69	92.00	4.69	4.69	4.69	3.75	5	6
class II	DR14	NS2A	VAHLALIAAFKVRPALLVSFIFR	1.42	94.47	2.37	2.37	2.37	0.00	0	2
class II	DR15	ancC	VSPFGGLKRLPAGLLLGH	1.29	91.43	0.00	0.00	0.00	0.00	3	2
class II	DR16	NS5	MWQLLYFHRRDLRLMANAICSSV	0.63	99.42	5.63	5.63	2.75	2.50	3	4
class II	DR17	NS4B	DIDLRPASAWAIYAALTTFITPAV	0.11	86.68	0.00	0.00	0.00	0.00	2	1
class II	DR18	NS5	LAIIKYTYQNKVVKVLRPAEKGKTVMD	1.53	97.37	1.58	1.58	3.16	4.74	0	1
class II	DR19	ancC	FLRFTAIKPSLGLINRWG	1.05	99.00	0.00	0.00	0.00	0.00	2	4
class II	DR20	E	SDLYYLTMNNKHWLVHK	0.00	97.23	0.00	0.00	0.00	0.00	3	4
class II	DR22	NS2A	KGSVKKNLPFVMALGLTAVRL	1.00	95.20	0.00	0.00	0.00	0.00	0	3
class II	DR23	preM	RVENWIFRNPGFALAAAAIAWLL	1.38	88.31	0.00	0.00	0.00	0.94	0	5
class II	DR25	E	IGTLLMWLGLNTKNGSISL	0.73	91.82	0.00	0.00	0.00	0.00	2	5
class II	DR26	NS4B	KSTLWEGSPNKYWNSSTATSLCNI	0.69	99.23	0.00	0.00	0.00	0.00	2	1
class II	DR27	NS3	NGVVIKNGSYVSAITQGRREE	1.50	96.14	0.00	0.00	0.00	0.00	1	5
class II	DR28	NS4B	RGSYLAGASLIYTVTRNAGLVKR	1.75	98.25	0.00	0.00	0.00	0.00	1	2
class II	DR29	NS5	EEPVLVQSYGWNIVRLKSGVDVFH	0.57	96.93	0.00	0.00	0.00	0.00	3	3
class II	DR30	NS3	PEIVREAIKTRLRTVILAPTRVVAAE	0.69	88.94	6.56	6.56	6.56	6.56	1	2
class II	DR32	NS2B	MREIILKVVLMTICGM	0.75	83.50	0.00	0.00	0.00	0.00	0	3
class II	DR33	NS1	NIMWRSVEGELNAILEEN	0.33	79.89	0.00	0.00	0.00	0.00	3	3
class II	DR34	NS4	KPLKHRAWNSFLVEDHGF	0.22	94.00	0.00	0.00	0.00	0.00	2	1
